# Distinctive, fine‐scale distribution of Eastern Caribbean sperm whale vocal clans reflects island fidelity rather than environmental variables

**DOI:** 10.1002/ece3.9449

**Published:** 2022-11-03

**Authors:** Felicia Vachon, Ana Eguiguren, Luke Rendell, Shane Gero, Hal Whitehead

**Affiliations:** ^1^ Department of Biology Dalhousie University Halifax Nova Scotia Canada; ^2^ School of Biology University of St. Andrews St. Andrews UK; ^3^ Department of Biology Carleton University Ottawa Ontario Canada

**Keywords:** Caribbean, cetacean, conservation, culture, habitat modeling, site fidelity, sperm whale

## Abstract

Environmental variables are often the primary drivers of species' distributions as they define their niche. However, individuals, or groups of individuals, may sometimes adopt a limited range within this larger suitable habitat as a result of social and cultural processes. This is the case for Eastern Caribbean sperm whales. While environmental variables are reasonably successful in describing the general distribution of sperm whales in the region, individuals from different cultural groups have distinct distributions around the Lesser Antilles islands. Using data collected over 2 years of dedicated surveys in the Eastern Caribbean, we conducted habitat modeling and habitat suitability analyses to investigate the mechanisms responsible for such fine‐scale distribution patterns. Vocal clan‐specific models were dramatically more successful at predicting distribution than general species models, showing how a failure to incorporate social factors can impede accurate predictions. Habitat variation between islands did not explain vocal clan distributions, suggesting that cultural group segregation in the Eastern Caribbean sperm whale is driven by traditions of site/island fidelity (most likely maintained through conformism and homophily) rather than habitat type specialization. Our results provide evidence for the key role of cultural knowledge in shaping habitat use of sperm whales within suitable environmental conditions and highlight the importance of cultural factors in shaping sperm whale ecology. We recommend that social and cultural information be incorporated into conservation and management as culture can segregate populations on fine spatial scales in the absence of environmental variability.

## Graphical abstract

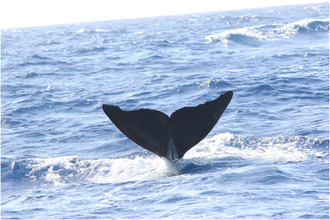

## 1 INTRODUCTION

It is not uncommon for species to only occupy a limited range within available suitable habitat. While environmental variables are often the primary driver of species distribution (as a failure to meet certain conditions will reduce fitness), social factors might also limit individuals' range within a wider suitable habitat. This is true for territorial species (e.g., wolves, *Canis lupus*—O'Neil et al., 2020, chimpanzees, *Pan troglodytes verus*—Herbinger et al., 2001), species that show site fidelity (e.g., fur seals, *Arctocephalus gazella*—Hoffman et al., 2006; reef fishes, *Thalassoma bifasciatum*—Warner, 1988), as well as prey type specialists (e.g., killer whales, *Orcinus orca*—Filatova et al., 2019) and habitat specialists (e.g., bottlenose dolphins, *Tursiops truncatus*—Kopps et al., 2014, elephants, *Loxodonta africana*—Fishlock et al., 2016). In cases of prey or habitat specialization, individuals learn to use, and can specialize on, prey or habitat features that are distributed differently from the prey or habitat features used by other members of the same species, thereby resulting in a heterogenous distribution. Territoriality, site fidelity, prey type specialization, and habitat specialization are often group‐level processes that can relate to kinship and/or social learning/culture (with culture defined as behavior or information shared within a community that is acquired from conspecifics through some form of social learning; Whitehead & Rendell, 2015). For instance, individuals might learn prey or habitat preferences via social learning within cultural groups as is the case in killer whale ecotypes (reviewed in Riesch et al., 2012) and/or via vertical transmission from parents as is the case with bottlenose dolphin “spongers” (Krützen et al., 2005).

However, although their effect on distribution can be quite dramatic, social factors such as the ones described above are rarely included in analyses relating to animal conservation. For instance, habitat models, which are a widespread tool in conservation as they allow for the identification of critical habitats for species' recovery and survival and can offer invaluable information regarding a population's health (Redfern et al., 2006), consider environmental variables in detail but rarely include cultural and social information (exceptions: Eguiguren et al., 2019; Filatova et al., 2019).

As more and more evidence suggests that culture is widespread in the animal kingdom (e.g., Whiten, 2017), there is increasing interest in the role of cultural transmission in determining species distribution (Brakes et al., 2021). This might be particularly important for species for which many group‐level behaviors are culturally transmitted, such as the sperm whale (*Physeter macrocephalus*) (e.g., Cantor et al., 2015).

Sperm whales are deep‐diving cetaceans that live in all of the world's oceans (Whitehead, 2003). They have a complex social structure in which females and calves live at lower latitudes year‐round in stable matrilineally‐based social units of about 10 members (Christal et al., 1998). Interactions between individuals and social units are then restricted to members of the same *vocal clan*, a higher‐order social structure defined by vocal dialect, that can occur in sympatry (Gero et al., 2016; Rendell & Whitehead, 2003). Vocal clans can include hundreds to tens of thousands of whales (Rendell & Whitehead, 2003), are identified by distinctive usage of stereotyped patterns of clicks called codas (Gero et al., 2016; Rendell & Whitehead, 2003), and have been documented worldwide (Amano et al., 2014; Amorim et al., 2020; Gero et al., 2016; Huijser et al., 2020; Rendell & Whitehead, 2003). Beyond acoustic differences, sperm whales from different vocal clans also display different social behaviors (Cantor & Whitehead, 2015), movement patterns (Vachon et al., 2022; Whitehead & Rendell, 2004), and distributions (Eguiguren et al., 2019; Vachon et al., 2022). Because vocal clans can live in sympatry and genetic variation is insufficient to explain this behavioral variation (Rendell et al., 2012), it is believed that vocal clans are cultural entities, with distinctive behaviors being socially learned largely within social units (Cantor et al., 2015). The existence of these culturally driven vocal clans has important implications for the behavior, ecology, and distribution of sperm whales, in a similar way to the *ecotypes* of killer whales (Riesch et al., 2012). Therefore, considering conservation metrics such as habitat use without accounting for culture might lead to misinterpretation as culture can alter behavior and distribution and subdivide populations in unexpected ways (Brakes et al., 2021; Whiten, 2017).

The population of sperm whales in the Eastern Caribbean has been extensively studied but, until recently, at a relatively small spatial scale (i.e., largely around a single island). Since 2005, The Dominica Sperm Whale Project (DSWP) has studied over 19 sperm whale social units around Dominica (Gero et al., 2014), gaining important insight on sperm whale social structure and behavior (Gero et al., 2014, 2016). In 2019 and 2020, we extended this research area and conducted surveys to include a wider range along the Lesser Antillean chain (from St. Kitts and Nevis to Grenada). From this, we gained insight into the way vocal clans influenced the spatial organization of the Eastern Caribbean sperm whale population (Vachon et al., 2022). Eastern Caribbean vocal clans (EC1 and EC2) appear to have very distinctive small‐scale distributions, with EC1 found predominantly around Dominica, Guadeloupe and St. Vincent and the Grenadines and EC2 found around the two central islands, St. Lucia and Martinique. This is not unheard of as sperm whale vocal clans in the Eastern Tropical Pacific have also been shown to have somewhat different distributions over a somewhat similar scale of 100s of kilometers (Eguiguren et al., 2019). However, the causes of such segregation have not been investigated until now.

We propose two competing hypotheses to explain vocal clan island segregation in the Eastern Caribbean. The first is habitat specialization, where islands vary in the amount of each vocal clan's preferred habitat type. In this case, foraging strategies specialized to specific habitat types could be driving the distribution of Eastern Caribbean sperm whale vocal clans. As sperm whales spend about 75% of their time foraging (Whitehead & Weilgart, 1991), differences in foraging strategies relating to environmental variation could lead to large differences in overall distribution. The second hypothesis is vocal clan‐specific traditions of island preferences that are arbitrary with respect to the habitat each island offers. This is akin to a classic study of mating site choice in blue head wrasse (*Thalassoma bifasciatum*) by Warner (1988) which first showed that preferred coral heads were in physical terms no different from unused ones, a pattern robust to translocation with persistent preferences socially maintained by traditions. In the case of Caribbean sperm whales, the different Lesser Antilles islands might be analogous to the different wrasse mating sites, with individuals from different vocal clans preferentially staying in the vicinity of certain islands for reasons of tradition (site/island fidelity) rather than specific physical features. While translocation experiments are not possible for sperm whales, we can ask whether clan‐specific habitat preferences map onto variation in the amount of preferred habitat across islands to understand whether these preferences are likely to be traditional or not.

Therefore, in this paper, we attempted to differentiate between habitat specialization and site/island fidelity by modeling sperm whale habitat use in the Eastern Caribbean, assessing the relative importance of island geography and habitat characteristics in predicting sperm whale presence by identifying important environmental variables for EC1 and EC2 whales independently, and testing whether the distribution of these variables varies significantly across the EC1 and EC2 “islands.” If Eastern Caribbean sperm whales are habitat specialists, we expect specific environmental variables to be closely linked with EC1 and EC2 distributions and there to be stark variation in at least some of these variables between EC1 and EC2 “islands.” On the contrary, if Eastern Caribbean sperm whale distribution is the result of culturally mediated island/site fidelity, we expect island vicinity to be a better predictor of EC1/EC2 sperm whale presence and environmental variables to not be significant factors in our models. Such an approach not only aims for a deeper understanding of a group‐living and cultural species' distribution and behavior, but also yields a novel approach to integrate into conservation policy.

## 2 METHODS

### 2.1 Field methods

**FIGURE 1:**
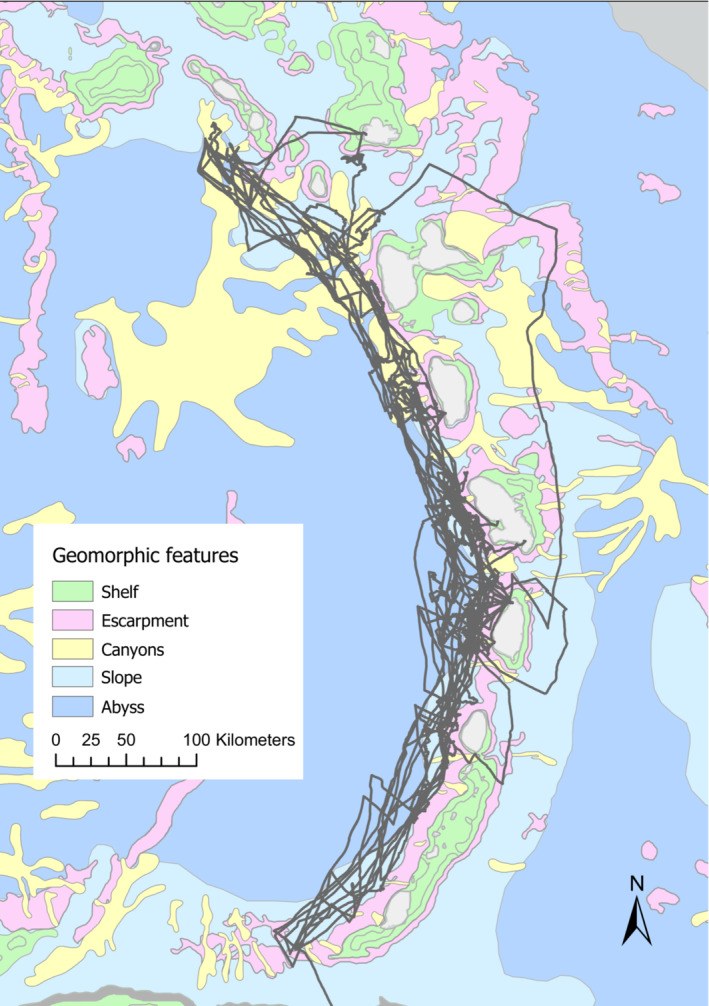
Map displaying the geomorphic features used to model sperm whale distribution in the Eastern Caribbean. Vessel tracks displayed in dark gray.

Data were collected between the months of February and April 2019 and January and March 2020 in the Eastern Caribbean. We surveyed sperm whale presence between the islands of St. Kitts and Nevis and Grenada along three transect lines (Leeward Inshore: 5–7 nautical miles from coast, Leeward Offshore: 15 nautical miles from coast and Windward Offshore: 5–7 nautical miles from shore) (Figure 1) from a 12 m auxiliary sailboat using a two‐element hydrophone array (two high‐frequency Magrec HPO3 elements with low‐cut filter set at 2 kHz) towed behind the vessel on a 100 m cable. Once encountered acoustically, female sperm whales were followed, using the towed hydrophone with the direction sensing software *Click Detector* on PAMGUARD, for hours to days. Codas to identify vocal clans were recorded via a Fireface UC or UMC202HD USB audio interface connected to a PC computer running software PAMGuard (Gillespie et al., 2009), sampling at 96 kHz and recording continuously during surveys. The GPS location of our research vessel was recorded on a GPS marine chart plotter (Standard Horizon in 2019 and Raymarine in 2020) every 5 min. Given that we could identify social units in real time using photo identification (see Gero et al., 2014), we intentionally spent more time with groups of whales for which we had little or no prior data and, if conditions allowed, stayed with unknown groups until we had repeats of multiple individual's flukes and had obtained at least 80 codas (this allowed for high confidence in identifying the vocal clan that the group belonged to) (Vachon et al., 2022).

### 2.2 Assigning GPS coordinates to vocal clans

All individuals identified on the same day were considered part of the same group if they had coordinated behavior and movement (Gero et al., 2014). Their codas were used to identify the group's vocal clan membership following methods by Hersh et al. (2021) (see Vachon et al., 2022). The GPS position of our research vessel was assigned to a vocal clan for the length of the encounter: From the time, we first heard the characteristic echolocation clicks of sperm whales until we could not hear them or chose to leave the whales due to weather or logistical constraints (Whitehead, 2003). We did not include encounters with Unit 12 (potential EC3 vocal clan) (Vachon et al., 2022) in this analysis as we have relatively little data regarding their distribution compared with EC1 and EC2. We considered GPS locations for which we had EC3 presence as the presence of sperm whales but did not include them as either EC1 or EC2 presence.

### 2.3 Habitat model variables

We included seven topographical variables (water depth—*Depth*, slope—*Slope*, distance to nearest submarine canyon—*Canyon*, distance to the escarpment—*Escarp*, distance to the abyss—*Abyss*, distance to shelf—*Shelf*, and distance to the center of the nearest channel between islands—*Channel*); six oceanographic variables (eastward current speed—*Ecurr*, northward current speed—*Ncurr*, zonal velocity variance—*Zvelv*, meridional velocity variance—*Mvelv*, inflow through the nearest channel—*Inflow*, and chlorophyll‐a concentration—*Chla*); and four general variables (latitude—*Lat*, longitude—*Long*, nearest island—*Island*, and whether the position is leeward or windward of the Lesser Antilles island chain—*Windward*)—for a total of 17 potential variables (Table S1), in our habitat models. These predictor variables were chosen as they were useful in describing sperm whale habitat in the Mediterranean and South Pacific and/or are thought to relate to the aggregation of sperm whale's prey, mesopelagic squid (Claro et al., 2020; Eguiguren et al., 2019; Pirotta et al., 2011).

Bathymetric data were obtained from the 2020 General Bathymetric Chart of the Oceans (https://www.gebco.net/data_and_products/gridded_bathymetry_data/) and extracted using ArcGIS. Slope was calculated from the GEBCO bathymetric layer using ArcGIS *Slope* tool. We used distance to geomorphic features canyon, escarpment, abyss, and shelf as predictor variables as in the habitat models of Claro et al. (2020). Geomorphic features' definitions and locations were obtained from Harris et al. (2014) via Blue Habitat (www.bluehabitats.org) (Figure 1). Oceanographic variables—eastward current speed, northward current speed, zonal velocity variance, and meridional velocity variance—were obtained from the NOAA drifter‐derived climatology of global near‐surface currents database (Laurindo et al., 2017). Chlorophyll‐a concentration was extracted from the NOAA visible infrared imaging radiometer suite (VIIRS) satellite data and averaged over the last 3 months prior to each data point to account for the lag between primary production and sperm whale prey availability (Jaquet, 1996). Measures of inflow through the nearest channel were obtained from Johns et al. (2002). The four general predictors were included to account for unexplained, or unaccounted, environmental variation in our data. Nearest island is a categorical variable that corresponds to the nearest island to a GPS point (in geodesic distance) and was extracted using the *Near* tool in ArcGIS. Windward/leeward is a binary variable that describes whether a GPS point is leeward, east, (N) or windward (Y) of the Lesser Antilles island chain.

The variables depth and slope were recorded at 0.004° spatial resolution; variables eastward current speed, northward current speed, zonal velocity variance, and meridional velocity variance were recorded at 0.25° resolution, and Chlorophyll‐a concentration was recorded at 0.036° resolution. As these resolutions are lower than that of our GPS coordinates, we used ArcGIS tools *Near* and *Spatial join* to extract the closest value for each variable to each GPS coordinate. We believe that the resolution at which those variables are available will not negatively affect our modeling approach as they have little small‐scale variability.

### 2.4 Habitat modeling

**FIGURE 2:**
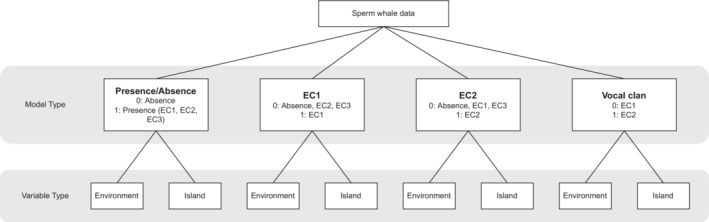
Summary of habitat modeling approach.

We used GPS fixes from the research vessel's chart plotter taken at 5 min intervals as our units of analysis. Each data point corresponds to specific coordinates at a certain time, along with whether sperm whales were acoustically encountered at that point and time, as well as the clan to which encountered whales belonged to (dataset available as supplementary material, Data S1). We fitted four different habitat model types (*Presence/Absence*, *EC1*, *EC2*, and *Vocal clan*) to our data using two independent sets of variables (*Environment* and *Island*) (Figure 2, defined below). Here, we describe each model type and the rationale for testing them across the two variable sets.

1. Presence/Absence: This model described the general distribution of sperm whales in the Lesser Antilles, regardless of vocal clan membership. The response variable was 0 for acoustic absence of sperm whale and 1 for acoustic presence of sperm whales. This allowed us to identify key variables for sperm whale habitat in the Lesser Antilles and assess whether modeling sperm whale distribution independently for each vocal clan resulted in a significant improvement in predictive accuracy.
2. EC1/EC2: These models described the distribution of sperm whales that were assigned to the EC1 and EC2 vocal clans, respectively. For the EC1 model, the response was 0 for the acoustic absence of sperm whales or the presence of EC2 and/or EC3 whales and 1 for the acoustic presence of EC1 whales. Conversely, for the EC2 model, the response was 1 for the acoustic presence of EC2 whales and 0 otherwise. These models allowed us to compare the performance of vocal clan‐specific habitat models to that of general habitat models (i.e., *Presence/Absence*) as well as identify important environmental variables for predicting the presence of EC1 and EC2 whales, respectively. These environmental variables were then used in our habitat suitability analysis (see below).
3. Vocal clan: This model was fitted to identify the variables that best distinguish between the presence of EC1 and EC2. The response was 0 for EC1 acoustic presence and 1 for EC2 acoustic presence. Here, a high predictive accuracy would suggest that individuals from different vocal clans prefer contrasting habitat model variables and, therefore, suggest an important contribution of social factors (i.e., vocal clan membership) to sperm whale distribution. The dataset used for the *Vocal clan* model was smaller than that for the *Presence/Absence*, *EC1*, and *EC2* models since we only used sperm whale presence data points (1 s in *Presence/Absence* model).

We tested these four habitat model types independently on two sets of variables: either a full set of environmental variables (*Environment* set), or nearest island variables (*Island* set), and compared their predictive performance. The *Island* set includes variables *Island* and *Windward*, while the environment set includes all remaining 15 environmental predictors and *Windward*. We expect models using the *Environment* variable set to perform much better than the ones using the *Island* variable set if sperm whales are habitat specialist and the opposite if patterns of distribution are driven by site/island fidelity. To avoid confusion, model names on their own (e.g., *Presence/Absence*) will refer to the models performed using the *Environment* variable set and models followed by “Island” will refer to the models performed using the *Island* variable set (e.g., *Presence/Absence Island*).

#### Modeling approach

Habitat models were fitted using generalized estimating equations (GEEs; Liang & Zeger, 1986), in which variables were used as predictors of sperm whale presence (*Presence/Absence*, *EC1*, and *EC2* models) or vocal clan membership (*Vocal clan* model), following Pirotta et al. (2011) and using package *geepack* in R (Højsgaard et al., 2005). This approach has been used in other cetacean distribution studies (e.g., Eguiguren et al., 2019; Pirotta et al., 2014; Tepsich et al., 2014) and is appropriate when data are recorded continuously along survey transects. We chose GEEs over other methods since they explicitly account for autocorrelation (Liang & Zeger, 1986). Data points were clumped into blocks that corresponded to sperm whale encounters. Under this framework, residuals are allowed to be correlated within blocks, but we assume independence between blocks. We used encounters as our blocking variable as it was successfully used in similar studies (Eguiguren et al., 2019; Pirotta et al., 2011), and we found this to be an appropriate grouping variable as the autocorrelation among data points eventually converged at 0 within each encounter (Figure S1). We modeled the relationship between variables and sperm whale presence as linear terms only, as including nonlinear relationships as in previous studies (Eguiguren et al., 2019; Pirotta et al., 2011) only slightly increased overall fit and predictive accuracy, at the cost of interpretability.

We structured our modeling approach into five steps (Figure S2, described below), which were repeated independently for the *Presence/Absence*, *EC1*, *EC2*, and *Vocal clan* models. R code available as supplementary material (Code S1).

##### Preparing variables

We looked at the variables' distributions and logged ones, which were highly skewed. All variables were then standardized by subtracting the mean and dividing by standard deviation to facilitate model convergence.

##### Removing collinearity

First, we calculated correlation coefficients between all pairs of predictor variables. Variables which had correlation coefficients above 0.4 were considered to be correlated and not included in the same model. From this, we built all possible combinations of uncorrelated predictors into potential models, which were then tested for multicollinearity by measuring the generalized variance inflation factor (GVIF) (*car* package in R). Models which had a predictor with a GVIF value above 3 were discarded, and all other potential models with GVIF values below 3 were used as the first step in backward stepwise selection.

##### Model selection

We used QIC (Pan, 2001), an extension of the Akaike Information Criterion (AIC) that applies to GEE models, to compare models using manual backward stepwise selection (package *MuMIn* in R, Bartoń, 2013). We started from all the potential combinations of uncorrelated predictors (step 2) and compared their QIC (ΔQIC) as we removed a single variable in turn. The model with the lowest QIC is then used as the starting model for the next step, repeating this procedure until the removal of any variable in the model leads to an increase in QIC. The higher the absolute value of ΔQIC between models, the larger the gap in their predictive performance. As such, we chose models with fewer variables if their ΔQIC was 10 or less from the original model to encourage variable removal. The variables within the final model are then ordered according to how much their removal increases QIC (from highest to lowest).

##### Model validation

The best models from step 3 were then further evaluated using leave‐one‐out cross‐validation where encounters were iteratively removed from the data. We compared the percentage of data points that were correctly assigned (predictive accuracy, Hastie et al., 2009) between the step 3 models to that of the same model minus one variable. If the predictive accuracy of models with fewer variables was higher than that of the original model, we removed that variable and started this process again until predictive accuracy was highest for the model from which we did not remove variables. This was done as stepwise selection using QIC can sometimes retain spurious variables (Pirotta et al., 2011).

Model performance was then assessed in terms of how well models fit the data (goodness of fit) by measuring the proportion of data points correctly assigned as presences or absences (or EC1/EC2 in the vocal clan models) using confusion matrices (Fielding & Bell, 1997). To transform model predictions from a range of probabilities to a binary (presence or absence), we used the point of maximum distance between the receiving operating characteristic (ROC) curve and the 45‐degree diagonal as the cut‐off probability, using the R package *ROCR* (Sing et al., 2005). Additionally, we measured model goodness of fit by calculating the area under the ROC curve (AUC), which also reflects overall model performance (Fielding & Bell, 1997).

We finally compared the performance metrics described between models with *Environment* variables and *Island* variables for each model type (*Presence/Absence*, *EC1*, *EC2*, and *Vocal clan*) to determine whether differences in distribution are driven primarily by habitat specialization or site/island fidelity.

##### Prediction maps

To display the results of our habitat models, we built prediction maps from the best post‐cross‐validation *Presence/Absence*, *EC1*, *EC2*, and *Vocal clan* models. Maps were built by importing our model predictions from R into ArcGIS Pro.

### 2.5 Habitat suitability analysis

To further establish whether vocal clans have distinct distributions as a result of habitat specialization or site/island traditions, we conducted a habitat suitability analysis for each Lesser Antilles island. This was done by creating a 0.1 degree grid of GPS points that extended 30 nautical miles offshore (representative of our effort, Figure 1) leeward of each island and assigning these points, and their corresponding environmental variable values, to the closest island. From this, we obtained a range of values for each environmental variable for each island which we could then compare between “EC1” and “EC2” islands. Only environmental variables that were part of the final *EC1* and/or *EC2* models were included in these analyses as they were the ones that were suggested to impact vocal clan distribution. We compared the environmental conditions between islands using *t*‐tests to test whether each environmental variable significantly differed between islands predominantly used by EC1 and islands predominantly used by EC2.

We expected environmental variables to be correlated to preferred islands if the environmental variables themselves are driving vocal clan distribution (e.g., EC1 whales prefer canyons and Dominica, Guadeloupe and St. Vincent have more canyons than St. Lucia and Martinique) and uncorrelated if vocal clans are distributed around different island due to site fidelity traditions (e.g., all islands have similar amounts of canyons but EC1 whale are only seen in Dominica, Guadeloupe, and St. Vincent).

## 3 RESULTS

Over our two field seasons (February to April 2019 and January to March 2020), we spent 107 days at sea (Figure 1). Sperm whales were located throughout the leeward transects, with higher concentrations found around Martinique, St. Lucia, and Dominica, but were not heard to windward of the islands. We had a total of 50 sperm whale encounters, 24 with EC1 groups, 22 with EC2 groups, five with an EC3 group, and one with both EC2 and EC3 (Vachon et al., 2022), from which we recorded 778 h of sperm whale vocalizations. Altogether, we obtained 26,776 GPS coordinate data points.

### 3.1 Habitat modeling

**FIGURE 3:**
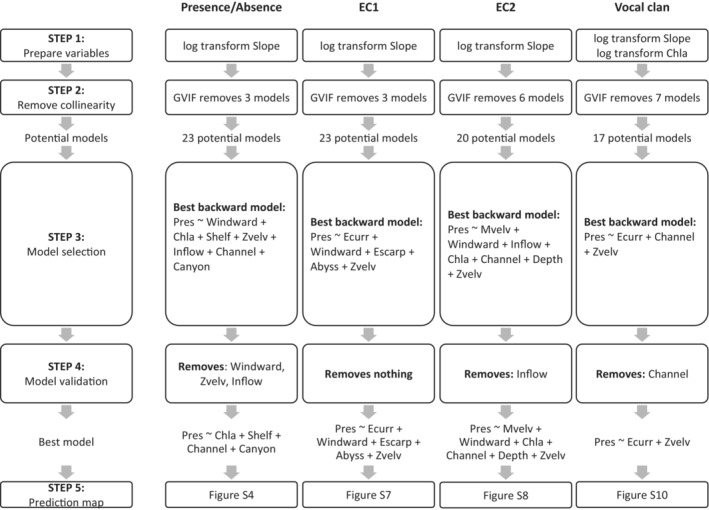
Summary of habitat modeling results for each habitat model at each step (*Environment* variable set).

Refer to Figure 3 for a full breakdown of the *Presence/Absence*, *EC1*, *EC2*, and *Vocal clan* habitat models at every selection step. Best pre‐cross‐validation and post‐cross‐validation habitat models, as well as corresponding results using the *Island* variable set, can be found in Table 1 and Table S2 with associated QIC, AUC, goodness of fit, and predictive accuracy. Below, we expand on general results from each model type.

**TABLE 1:** Best variable combinations for each model type with associated QIC, ΔQIC, AUC, goodness of fit, and predictive accuracy (post‐stepwise cross‐validation)

Model type	Variable set		QIC	ΔQIC	AUC	Goodness of fit (%)	Predictive accuracy (±SE)
Presence/Absence	Env	Chla + Shelf + Channel + Canyon	32,966.3	2281.4	0.71	69.8	50.62% (±0.02)
	Island	Windward + Island	30,684.9	‐	0.69	65.8	59.61% (±0.04)
EC1	Env	Ecurr + Windward + Escarp + Abyss + Zvelv	19,006.3	3115.5	0.79	77.1	56.65% (±0.03)
	Island	Windward + Island	15,890.8	‐	0.86	72.9	72.05% (±0.04)
EC2	Env	Mvelv + Windward + Chla + Channel + Depth + Zvelv	16,522.2	501.4	0.86	75.35	57.73% (±0.02)
	Island	Windward + Island	16,020.8	‐	0.83	73.2	62.27% (±0.04)
Vocal clan	Env	Ecurr + Zvelv	6152.1	5033.8	0.92	92.0	49.7% (±0.05)
	Island	Island	1118.3	‐	0.99	96.5	76.8% (±0.14)

*Note*: Using habitat models and habitat suitability analyses, we present and discuss a remarkable and unexpected pattern in the distribution of Eastern Caribbean sperm whales. Unlike their Pacific conspecifics, Eastern Caribbean sperm whales have short‐range movements and display island fidelity across multiple years. Such fine‐scale distribution appears to be culturally driven with different cultural groups (called vocal clans) occupying distinctive islands along the Lesser Antilles as a result of traditions of site fidelity rather than environmental variation.

#### 3.1.1 Presence/Absence model

This model had 50.62% predictive accuracy and 69.8% goodness of fit in determining sperm whale presence, regardless of vocal clan, using environmental variables. Sperm whales were more often encountered in areas with low chlorophyll‐a concentration, close to the continental shelf, relatively close to between‐island channels and further away from canyons (Figure S3). The negative correlation between presence and chlorophyll‐a concentration could be caused by the relatively low chlorophyll‐a concentrations across the Lesser Antilles chain, spatial lag between Windward productivity and leeward biomass or the temporal lag between primary productivity and cephalopod biomass (Jaquet, 1996; Pirotta et al., 2011), although we tried to account for this by considering chlorophyll‐a concentration over the last 3 months as in Eguiguren et al. (2019). The final *Presence/Absence Island* model (Pres ~ *Windward* + *Island*) performed better than the *Presence/Absence* model (Pres ~ *Chla* + *Shelf* + *Channel* + *Canyon*) with ΔQIC of 2281.4. The *Presence/Absence Island* model had 59.61% predictive accuracy and 65.8% goodness of fit in determining sperm whale presence and suggests that more sperm whales occupy the waters off the central islands of Dominica and Martinique (Figure S4), for reasons not fully explained by the environmental variables that we considered.

#### 3.1.2 EC1 and EC2 models

Modeling sperm whale distribution independently for EC1 and EC2 increased model predictive accuracy, goodness of fit and lowered QIC for both the models using environment and island variables (Table 1).

EC1 whales prefer areas of low eastward current speed, low zonal velocity variance, within the escarpment designation, away from the abyss, leeward of the Lesser Antilles chain (Figure S5). By contrast, EC2 whales prefer areas with high meridional velocity variance, low chlorophyll‐a concentration, deeper in the ocean, and low zonal velocity variance, closer to channels leeward of the Lesser Antilles chain (Figure S6). Unsurprisingly, variable *Windward* was important for both the *EC1* and the *EC2* models since sperm whales were not heard windward of the island chain. This result should be viewed cautiously since the leeward side of the island chain was much more extensively surveyed than the windward side (Figure 1). Zonal velocity variance (*Zvelv*) was also important for both models with EC1 sperm whales encountered in areas of high zonal velocity variance and EC2 sperm whales encountered in areas with low zonal velocity variance (Figures S5 and S6).

The best *EC1* model (Pres ~ *Ecurr* + *Windward* + *Escarp* + *Abyss* + *Zvelv*) and the best *EC2* model (Pres ~ *Mvelv* + *Windward* + *Chla* + *Channel* + *Depth* + *Zvelv*) performed worse than the *EC1 Island* (Pres ~ *Windward* + *Island*) and *EC2 Island* (Pres ~ *Windward* + *Island*) models with respective ΔQIC of 3115.5 and 501.4. According to our prediction maps, we expect EC1 sperm whales to aggregate near Dominica, Guadeloupe, St. Vincent and the Grenadines and St.Kitts and Nevis; and EC2 sperm whales to aggregate near St.Lucia and Martinique (Figures S7 and S8). Such predictions not only reflect, as expected, the field observations that were used to construct this model (Vachon et al., 2022), but also results from the long‐term research off Dominica by the DSWP, with EC2 groups seldom encountered off Dominica (only 2.5% of photo identification encounters; Gero et al., 2016; Vachon et al., 2022).

#### 3.1.3 Vocal clan model

This model had great accuracy in distinguishing between EC1 and EC2 vocal clan distribution using both the *Environment* and *Island* variable sets (92% and 96.5% goodness of fit, and 49.7% and 76.8% predictive accuracy). EC1 whales were more often encountered in areas of low eastward current speed and high zonal velocity variance, while EC2 whales were more often encountered in areas of high eastward current speed and low zonal velocity variance (Figure S9).

The *Vocal clan Island* model (Pres ~ *Windward* + *Island*) performed better than the *Vocal clan* model (Pres ~ *Ecurr* + *Zvelv*) with ΔQIC of 5033.8, and EC1 whales predominantly near the islands of Dominica, Guadeloupe and St. Vincent and the Grenadines and EC2 predominantly near St. Lucia and Martinique (Figure S10).

### 3.2 Habitat suitability

**FIGURE 4:**
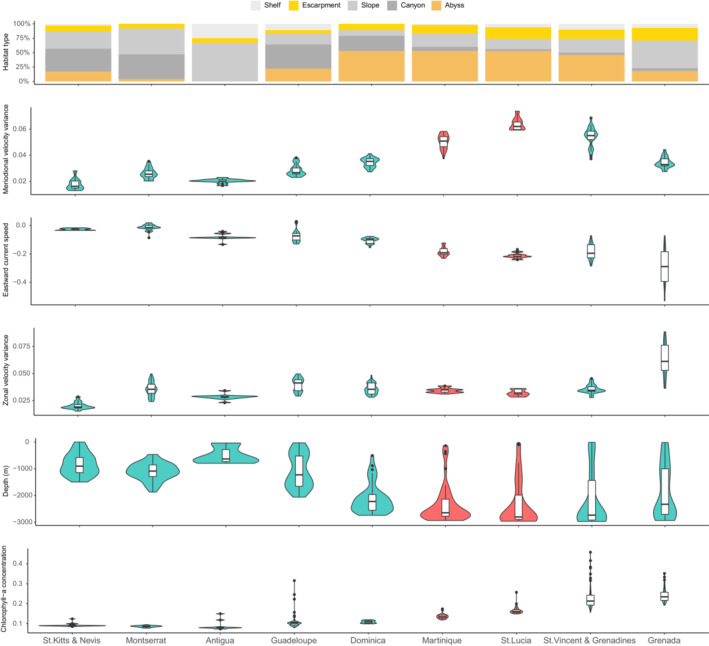
Habitat suitability of EC1 (aquamarine) and EC2 (red) islands according to significant environmental variable range within a 0.1 degree grid extending 30 nautical miles leeward of each island. No significant differences in variable values between EC1 and EC2 islands.

The lower QIC and higher predictive accuracy of the *EC1 Island*, *EC2 Island*, and *Vocal clan Island* models (Table 1) suggest that vocal clan distribution might be better explained by site/island fidelity than the use of specific habitat variables. Our habitat suitability results also corroborated this conclusion as the environmental variables that were considered significant predictors of EC1 and EC2 presence in the *EC1* and *EC2* models did not significantly differ between EC1 and EC2 islands, apart from *Abyss* and *Depth* (*t* = −4.01, *p*‐value = .007 and *t* = 3.68, *p*‐value = .010, respectively; Figure 4). Altogether this suggests that sperm whales from different vocal clans do not use different islands because they have a unique, or significantly different, selection of physical habitat properties.

Similar results were obtained if we only used surveyed grid points rather than the extrapolated 30 nautical mile offshore 0.1 degree grid to carry out this analysis (Figure S11).

## 4 DISCUSSION

In this study, we attempted to test the competing hypotheses of habitat specialization and traditional site/island fidelity in explaining the stark differentiation in EC1 and EC2 vocal clan distributions in the Eastern Caribbean. Our results suggest that site/island fidelity, rather than environmental variation, is the main driver of sperm whale distribution in the Lesser Antilles, with different processes operating at the species and vocal clan levels.

At the species level, sperm whales use areas that are close to the continental shelf and channels (*Presence/Absence* model). Such correlations between sperm whale distribution and topography have been documented for sperm whales elsewhere (e.g., Claro et al., 2020; Pirotta et al., 2011; Wong & Whitehead, 2014) and most probably reflect food availability as vertical water movement associated with sloped areas likely promotes primary and secondary productivity (Tynan et al., 2005). However, such coarse models fail to capture the variability created by differences in unit movement, clan membership, and foraging success at finer spatial scales (as reported by Jaquet & Whitehead, 1996 in the South Pacific) and seemed to be impacted, even at this scale, by the whales' bias toward certain islands with the *Presence/Absence Island* model performing better than the *Presence/Absence* (Table 1).

The dramatic increase in the performance of vocal clan‐specific models over a general species presence model is one of the most striking results of our study. The preference of the EC2 vocal clan for St. Lucia and Martinique and the EC1 vocal clan for Dominica, Guadeloupe, and St. Vincent and the Grenadines does not relate to environmental variables, as they do not significantly or substantially differ across islands (Figure 4), but rather seem to be caused by site/island fidelity with the *EC1 Island*, *EC2 Island*, and *Vocal clan Islands* models performing much better than their counterparts (Table 1). In this case, culture, via conformism and homophily to island preference traditions, would act as a barrier to population mixture (e.g., Centola et al., 2007; Riesch et al., 2012). We suggest that individual sperm whales stay in the vicinity of specific islands because those are the islands where they were raised, where they learned to forage, where their close associates and family members can be encountered, and where they can avoid interactions with members of other vocal clans. Conformism and homophily have already been reported in Eastern Caribbean sperm whales with highly stereotypical vocal repertoires (conformity, Konrad et al., 2018) and individuals exclusively associating with members of their own vocal clan (homophily, Gero et al., 2016). It is also not surprising that individual sperm whales could learn island preferences from other members of their social units as other behaviors are culturally maintained within vocal clans (e.g., social vocalizations [Gero et al., 2016; Rendell & Whitehead, 2003], dive synchrony [Cantor & Whitehead, 2015], movement patterns [Vachon et al., 2022; Whitehead & Rendell, 2004], and social structures [Cantor & Whitehead, 2015]) and since cultural transmission has been suggested as the most likely mechanism for the emergence of vocal clans themselves (Cantor et al., 2015).

### 4.1 Limitations

This study is limited in its temporal scope. While EC1 and EC2 distribution patterns were stable over the 2 years of this study, and while they appear to have been stable since 2005 (Gero et al., 2014, 2016; Vachon et al., 2022), shifts could still occur over longer timescales, as it did in the Galapagos (Cantor et al., 2016). However, while the location of Eastern Caribbean vocal clans might change in future, the mechanisms responsible for their spatial segregation are likely to remain the same. This study might also be limited by the environmental variables that were included in habitat models. However, this is unlikely as we cover a wide array of environmental variable types (geomorphic features, oceanographic processes, and biological processes), including variables that were previously considered important for sperm whale habitat (e.g., Claro et al., 2020; Eguiguren et al., 2019; Pirotta et al., 2011) and environmental variables are rarely totally uncorrelated. Future research could investigate sperm whale prey density (e.g., from squid species survey and scat samples) and examine how prey density varies with the presence of different vocal clans and/or the proximity of different islands. Measures of sperm whale prey density remain undocumented in the Lesser Antilles.

### 4.2 Implications for conservation

The performance of our habitat models was greatly improved by the inclusion of a cultural indicator. We suggest that the low predictive accuracy of our *Presence/Absence* model is caused by confounding variables across vocal clans, something that could also explain why other sperm whale habitat models sometimes fail to reach high predictive accuracy when compared to other cetacean species (e.g., Claro et al., 2020; Tepsich et al., 2014).

Our results highlight how cultural factors can lead to important, management‐relevant variations in the way population segments use any given habitat, even at relatively small geographic scales for a large, highly mobile, and pelagic animal. In this case, traditions of site/island fidelity appear to be a more important determinant of sperm whale distribution within suitable habitat than are environmental variables. Adding this cultural lens, not only allowed for a better understanding of population structure, but also habitat use—two crucial variables in conservation and management.

Like many other populations, Eastern Caribbean sperm whales are now facing unprecedented anthropogenic threats related to global warming, increased ocean noise, and other human activities (e.g., Weilgart, 2007; Whitehead et al., 2008). Sperm whales studied off Dominica (predominantly EC1 units) were declining at a 4.5%/year rate between 2010 and 2015 (Gero & Whitehead, 2016), and the same might be true for sperm whales inhabiting the other Lesser Antilles islands. Under these circumstances, it is critical to build detailed habitat models which capture both important cultural and environmental variables. These habitat models can not only be used to help protect the population as a whole, but also identify areas of high importance for each cultural group. This aligns with recent conservation shift away from solely genetic diversity to the incorporation of cultural diversity as an important component of populations' health (Brakes et al., 2021) and supports the recognition of sperm whale vocal clans as independent evolutionarily significant units (ESU) for conservation and management.

### 4.3 Implications for sperm whale ecology/psychology

This study aimed at incorporating both environmental and cultural variability into the commonly used ecological and conservation approach of habitat modeling. By independently modeling vocal clan distribution, we were able to gain a more detailed insight into sperm whale population structure, the mechanisms responsible for their distribution, and greatly increase habitat model accuracy. Our results suggest that sperm whale habitat use in the Eastern Caribbean is predominantly shaped by cultural information rather than environmental cues. Given the matrilineal social structure of these groups, this not only highlights the importance of older females, mothers, aunts, and grandmothers as repositories of knowledge within social units and vocal clans (as is the case in elephants—McComb et al., 2001), but also implies that sperm whales are able to recognize and communicate fine‐scale cultural boundaries in the absence of physical barriers or environmental gradients. Over long timescales, these boundaries are unlikely to be impermeable (as few EC2 encounters have been documented in Dominica; Gero et al., 2016) and might change (e.g., Eastern Tropical Pacific vocal clan turnover—Cantor et al., 2016), but nonetheless remain culturally driven. As such, our findings have implications beyond the Eastern Caribbean, and beyond sperm whales, to our understanding of cultural species. It is crucial to assess the distribution, and behavior, of complex species in all their complexity (genetic, environmental, cultural, and their intersections) to properly conserve and understand them.

## AUTHOR CONTRIBUTIONS

**Shane Gero:** Methodology (supporting); writing – review and editing (equal). **Luke Rendell:** Funding acquisition (lead); investigation (supporting); writing – review and editing (equal). **Hal Whitehead:** Funding acquisition (supporting); investigation (supporting); supervision (lead); writing – review and editing (equal). **Felicia Vachon:** Conceptualization (lead); data curation (lead); formal analysis (lead); funding acquisition (supporting); investigation (lead); methodology (lead); project administration (lead); visualization (lead); writing – original draft (lead). **Ana Eguiguren:** Formal analysis (supporting); investigation (supporting); methodology (supporting); resources (equal); writing – review and editing (equal).

## CONFLICT OF INTEREST

We have no conflict of interest to disclose.

## Supporting information


Appendix S1

Data S1

Code S1

## Data Availability

Data are available as supplementary material and on Dryad at: https://doi.org/10.5061/dryad.mcvdnck4c.
