# Supplementary material for: Distinctive, fine‐scale distribution of Eastern Caribbean sperm whale vocal clans reflects island fidelity rather than environmental variables
Source: Ecol Evol. 2022 Nov 3;12(11):e9449. doi: 10.1002/ece3.9449 (PMC9631323; doi:10.1002/ece3.9449)
Supplement: Supplementary file 1 — Appendix S1 [file ECE3-12-e9449-s002.docx]

| 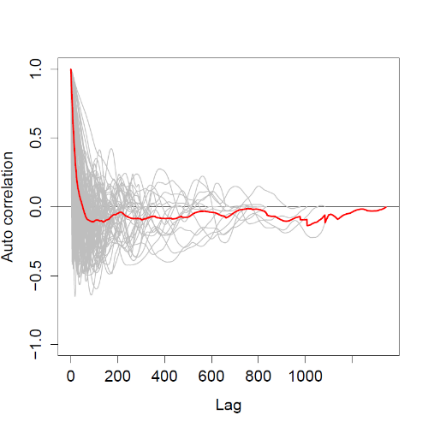  A1  A2  B1  B2 | 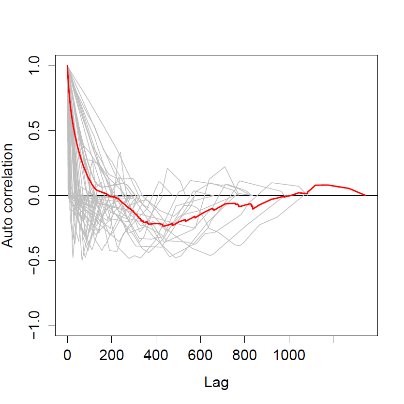 |
| --- | --- |
| 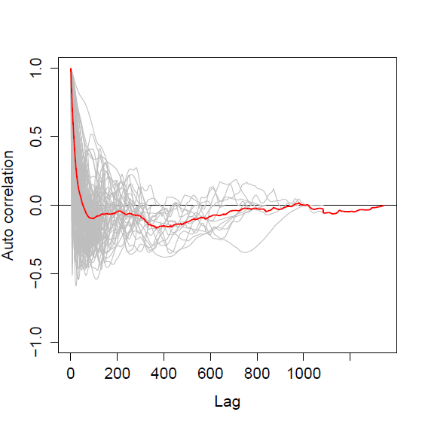 | 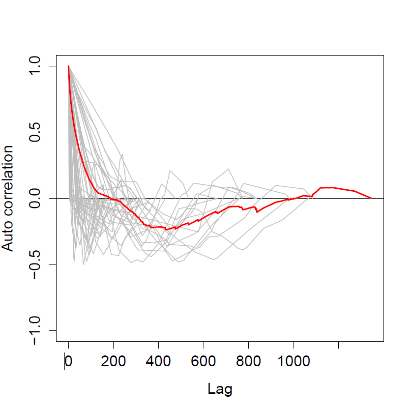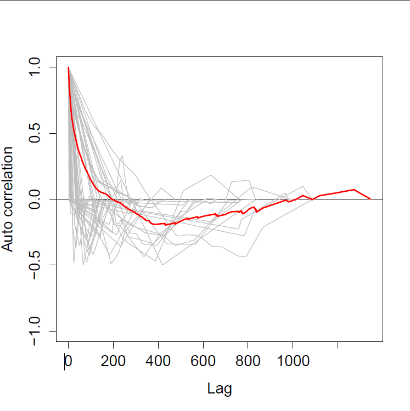 |
| 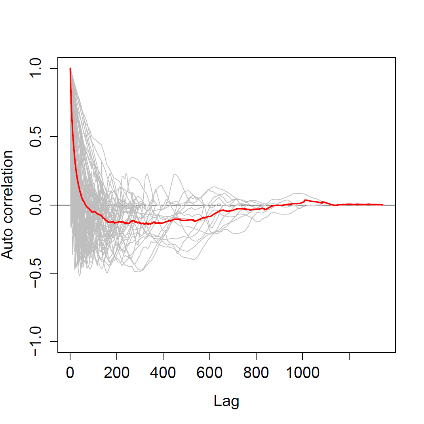 | C2  C1 |
| 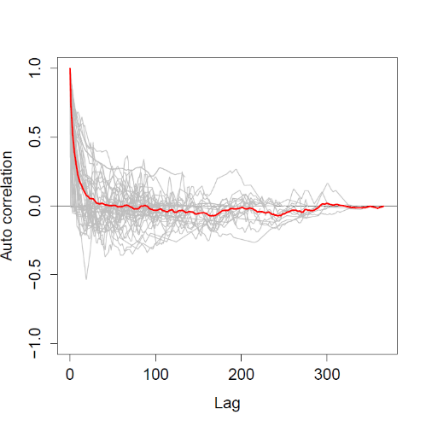  D1 | 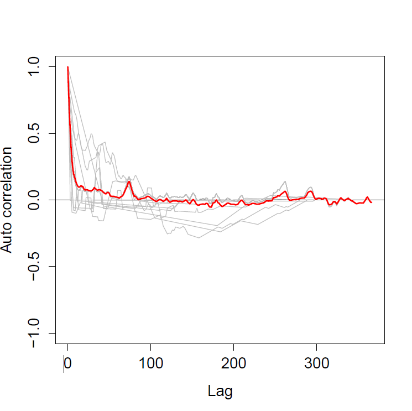  D2 |

Figure S1. Auto correlation function (ACF) plots of residuals for the final models of the *Presence/Absence* (A), *EC1* (B), *EC2* (C) and *Vocal clan* (D) habitat models using the *Environment* (1) variable set and *Island* (2) variable set. Plots rapidly converge to zero, which suggests that encounter is an appropriate blocking variable.

Figure S2. Summary diagram of habitat modelling steps. These were repeated independently for the *Presence/Absence*, *EC1*, *EC2* and *Vocal clan* model using the *Environment variable* set.


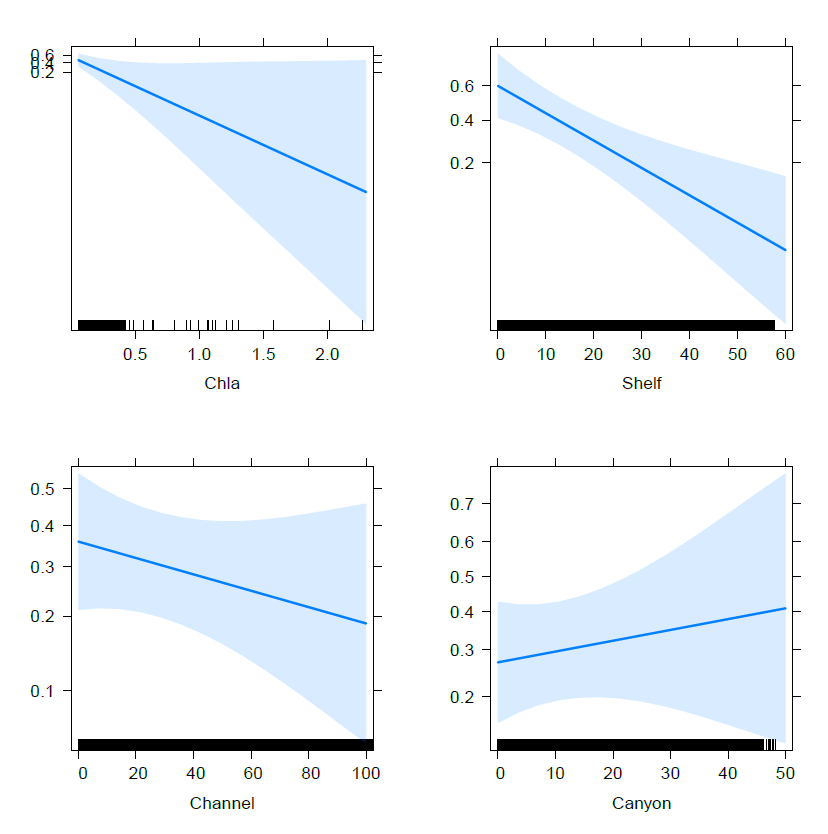


Figure S3. Effect plots of variables from the best *Presence/Absence* model (Pres ~ *Chla* + *Shelf* + *Channel* + *Canyon*).


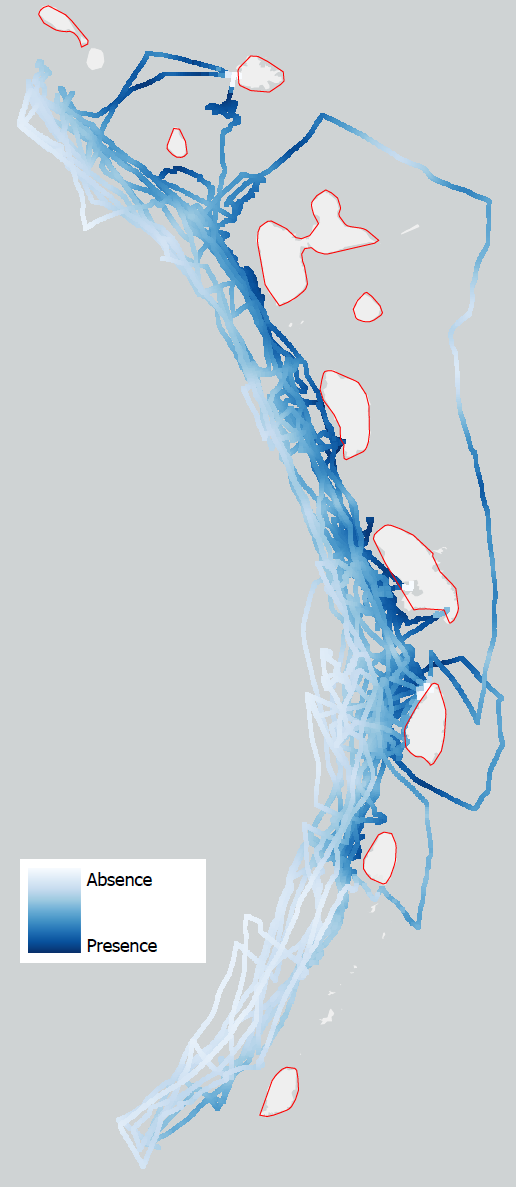

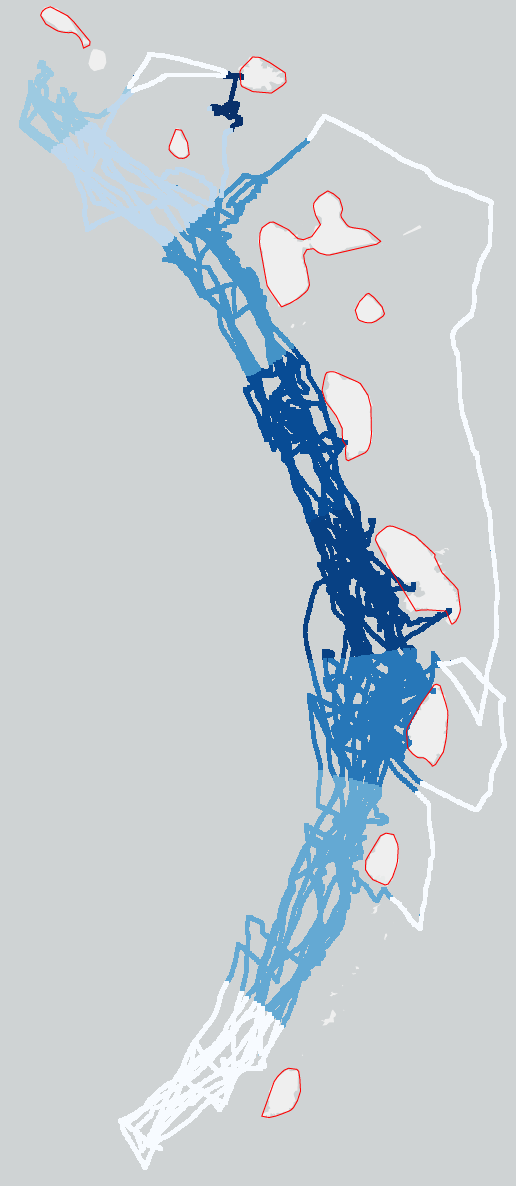


A

B

Figure S4. Prediction map of Eastern Caribbean sperm whale presence in the Lesser Antilles (*Presence/Absence* model). A) *Environment* variable set (Pres ~ *Chla + Shelf + Channel + Canyon*) B) *Island* variable set (Pres ~ *Windward* + *Island*).


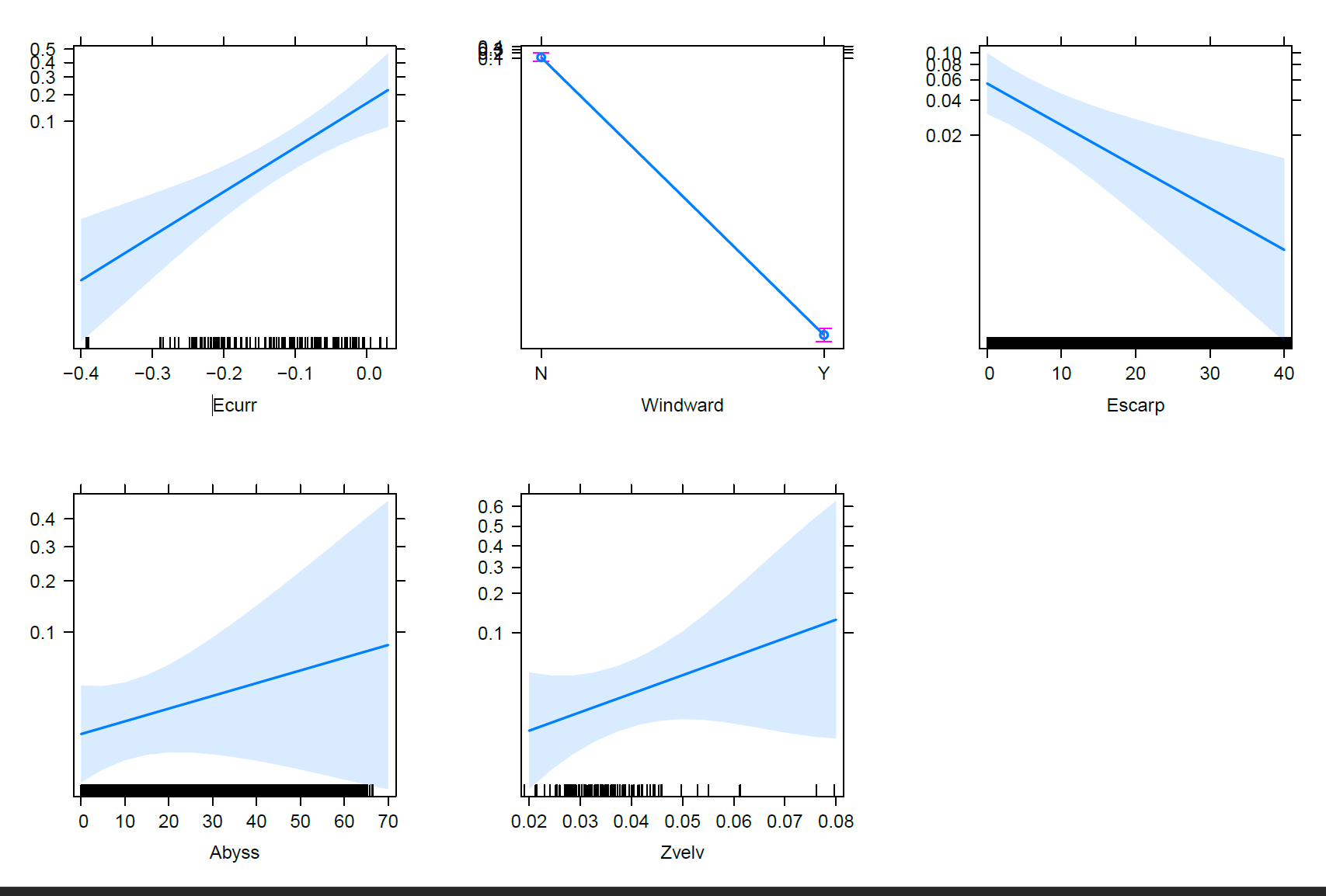


Figure S5. Effect plots of variables from the best *EC1* model (Pres ~ *Ecurr* + *Windward* + *Escarp* + *Abyss* + *Zvelv*).


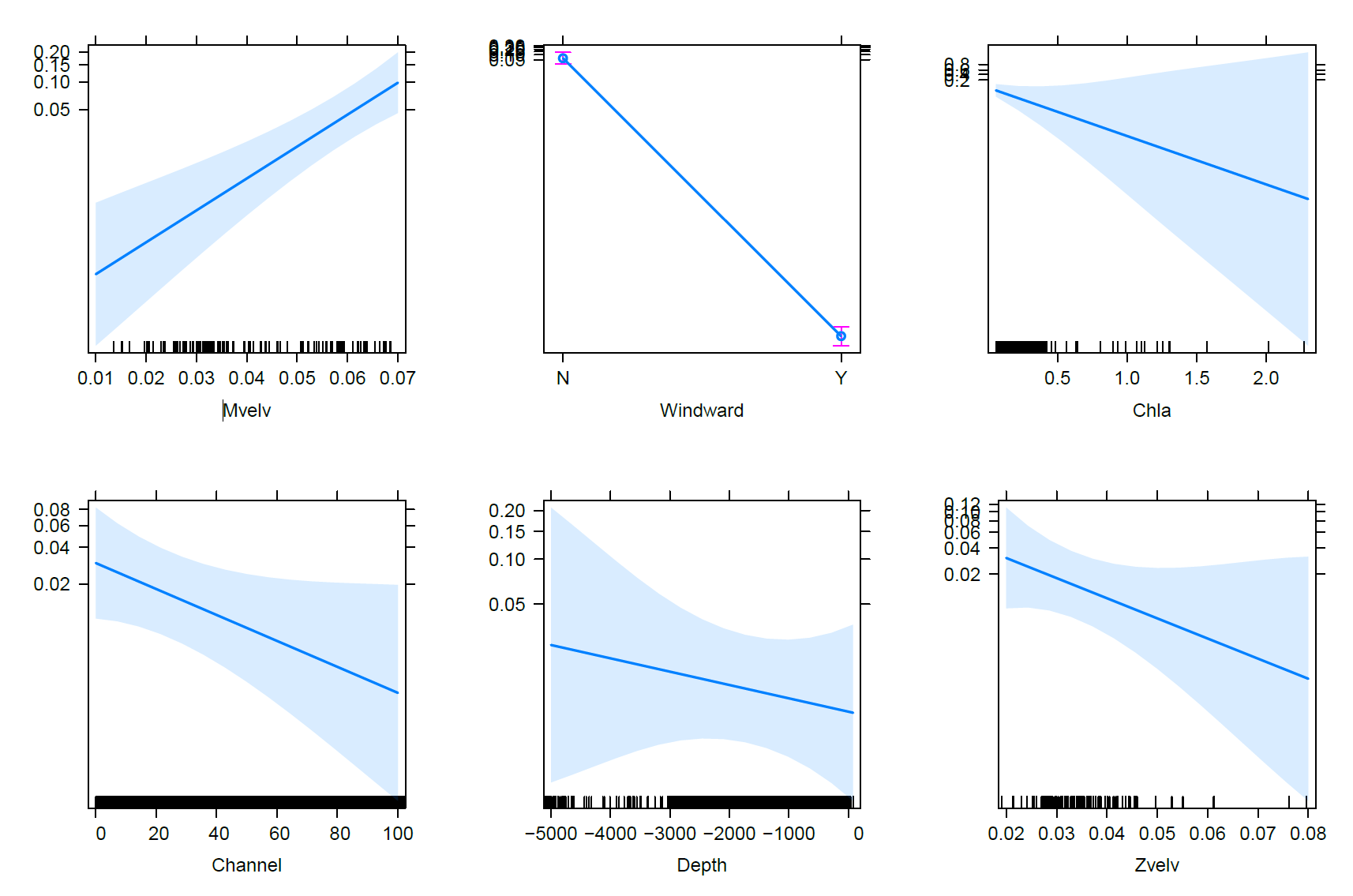


Figure S6. Effect plots of variables in the best *EC2* model (Pres ~ *Mvelv* + *Windward* + *Chla* + *Channel* + *Depth* + *Zvelv*).


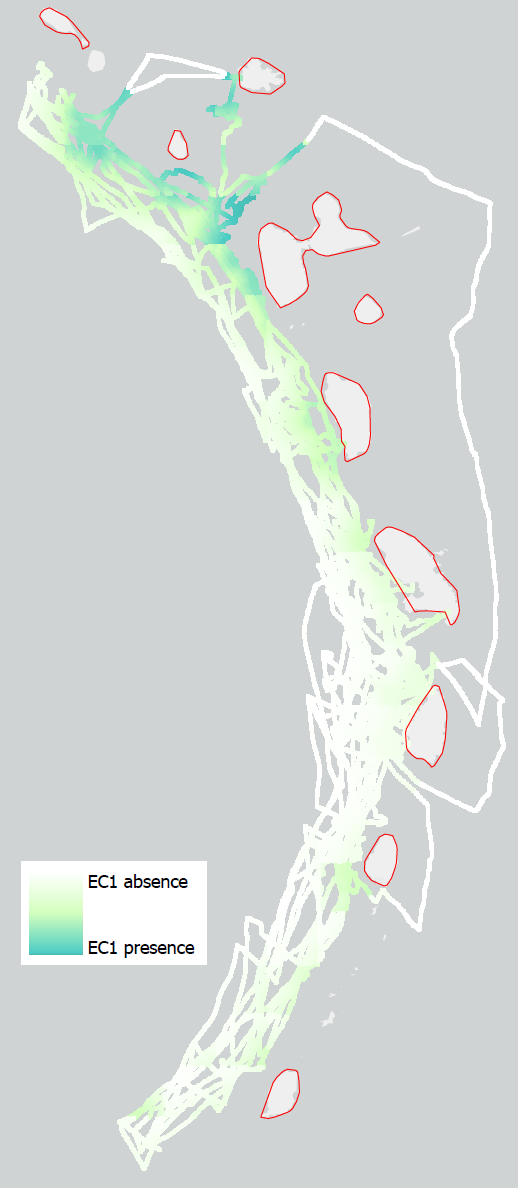

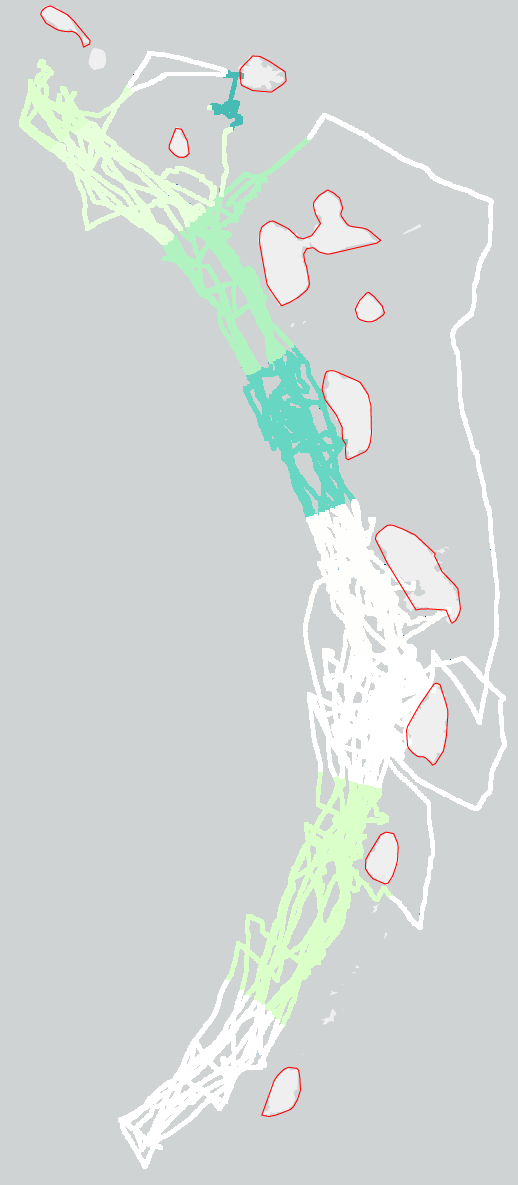


B

A

Figure S7. Prediction maps of EC1 sperm whale presence in the Lesser Antilles using the A) *Environment* variable set (Pres ~ *Ecurr* + *Windward* + *Escarp* + *Abyss* + *Zvelv*) B) *Island* variable set (Pres ~ *Windward* + *Island*).


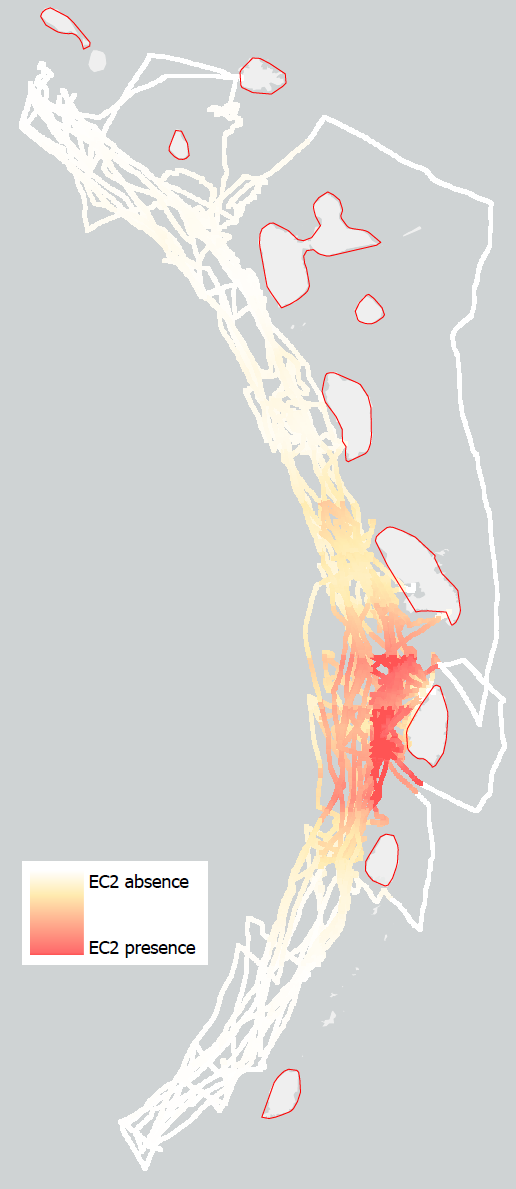

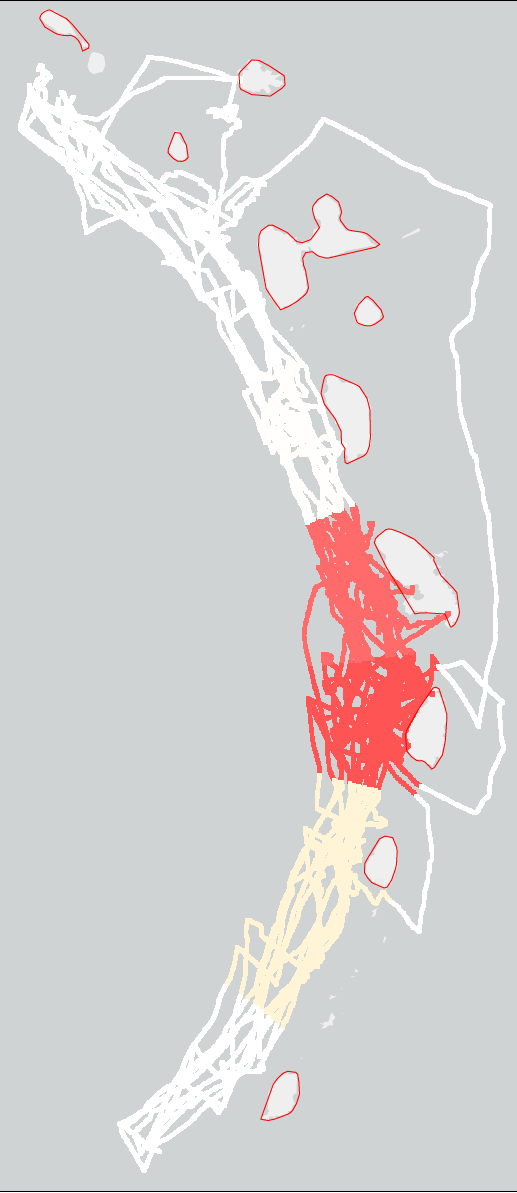


B

A

Figure S8. Prediction maps of EC2 sperm whale presence in the Lesser Antilles using the A) *Environment* variable set Pres ~ *Mvelv* + *Windward* + *Chla* + *Channel* + *Depth* + *Zvelv*) B) *Island* variable set (Pres ~ *Windward* + *Island*).


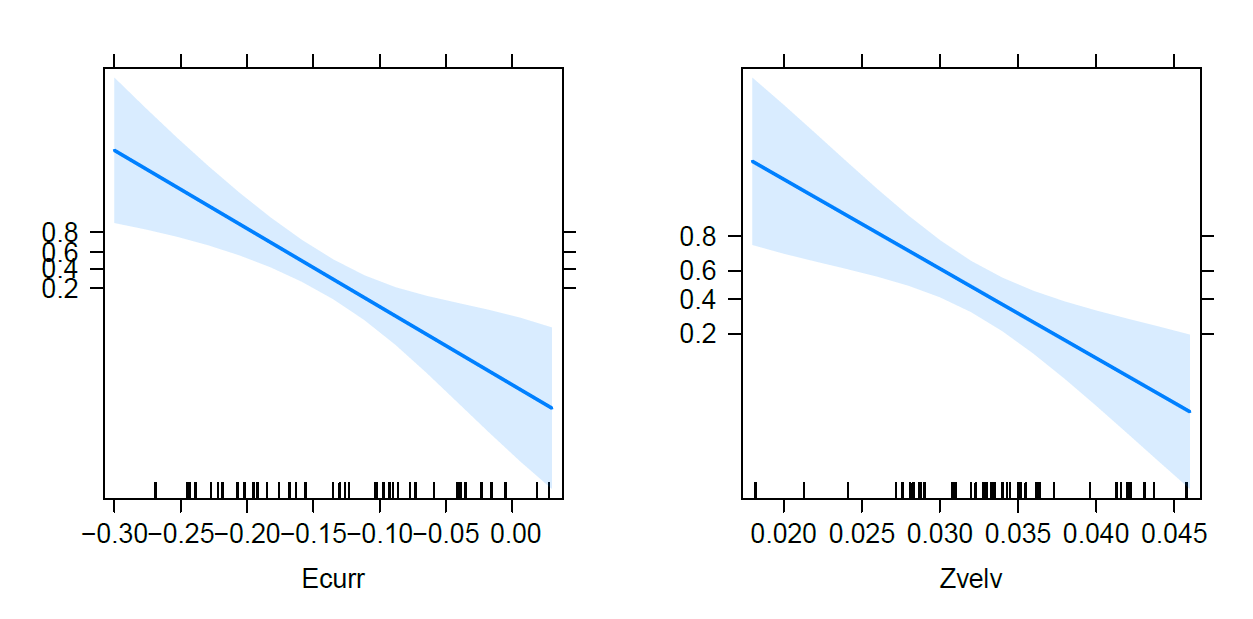


Figure S9. Effect plots of variables from the best *Vocal clan* model (Pres ~ *Ecurr* + *Zvelv*).


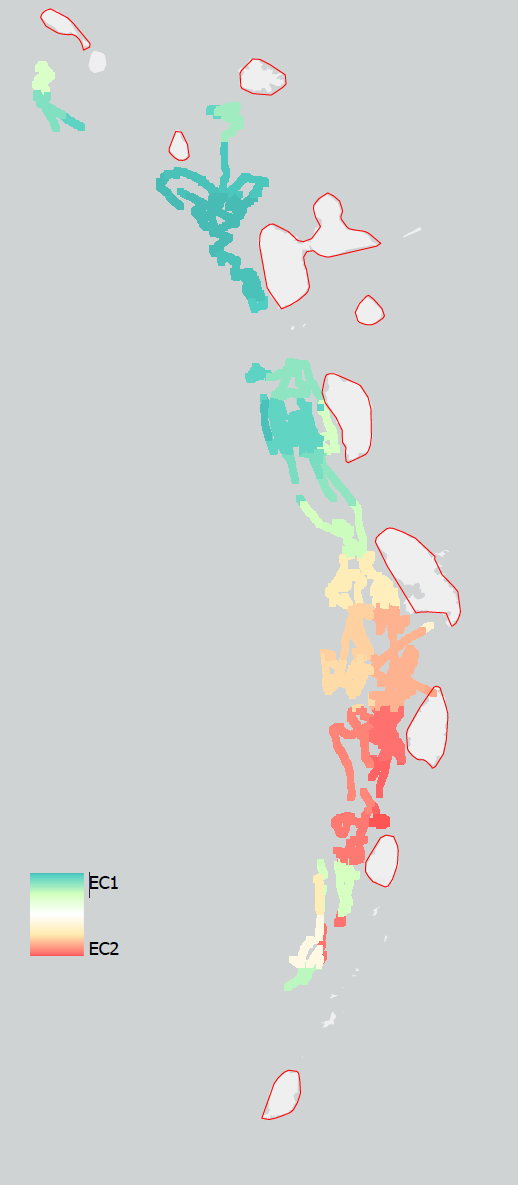

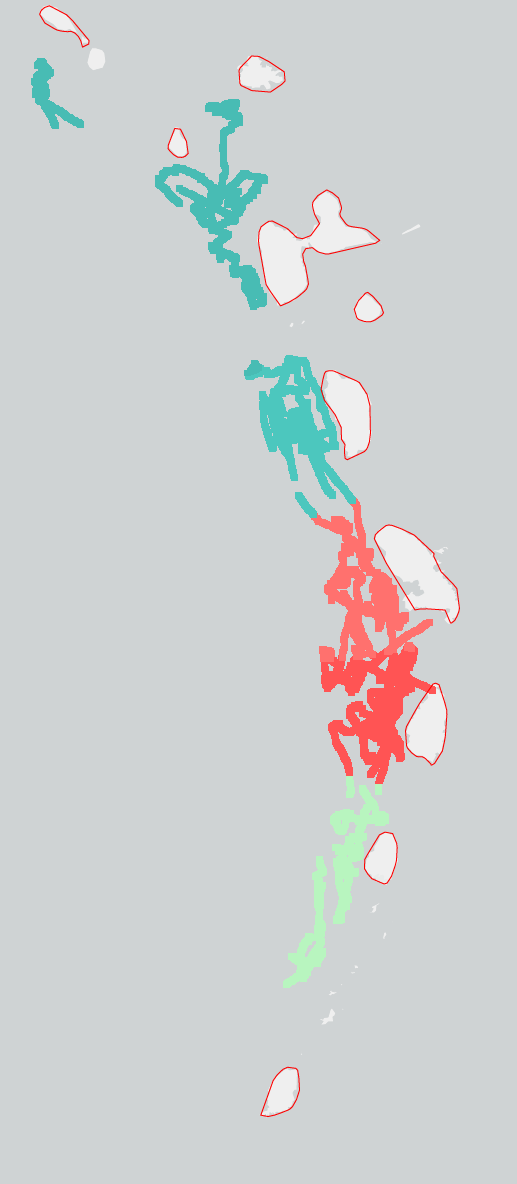


B

A

Figure S10. Prediction map of EC1 versus EC2 distribution in the Lesser Antilles (*Vocal clan* model). A) *Environment* variable set (Pres ~ *Ecurr* + *Zvelv*) B) *Island* variable set (Pres ~ *Island*).


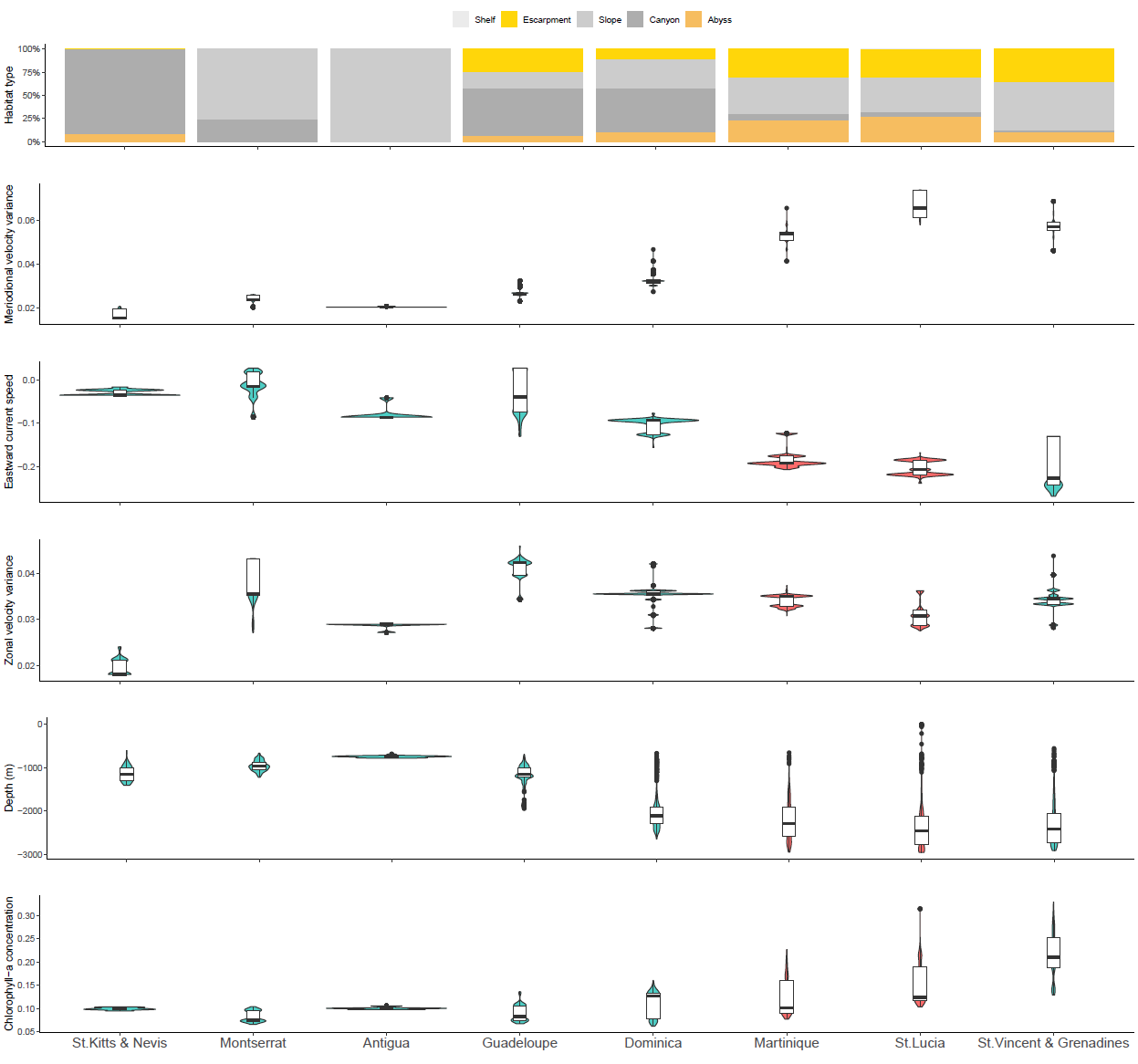


Figure S11. Habitat suitability of EC1 (aquamarine) and EC2 (red) islands according to significant environmental variable range within sperm whale presence data points. No significant differences in variable values between EC1 and EC2 islands.

Table S1. Description of predictor variables used in habitat models. Spatial resolution is in degrees of latitude.

| Name | Referred in model as | Description | Spatial resolution | Source |
| --- | --- | --- | --- | --- |
| Latitude | *Lat* | Latitude | NA | Chart plotter |
| Longitude | *Long* | Longitude | NA | Chart plotter |
| Depth | *Depth* | Bathymetry of the ocean | 0.004° | GEBCO 2020 |
| Slope | *Slope* | Steepness of the ocean floor calculated from depth in ArcGIS | 0.004° | NA |
| Distance to nearest canyon | *Canyon* | Distance to the nearest geomorphic feature canyon | NA | Harris et al. 2014 |
| Distance to escarpment | *Escarp* | Distance to the nearest geomorphic feature escarpment | NA | Harris et al. 2014 |
| Distance to abyss | *Abyss* | Distance to the nearest geomorphic feature abyss | NA | Harris et al. 2014 |
| Distance to shelf | *Shelf* | Distance to the continental shelf | NA | Harris et al. 2014 |
| Eastward current speed | *Ecurr* | Eastward speed of near surface currents | 0.25° | Laurindo et al. 2017 |
| Northward current speed | *Ncurr* | Northward speed of near surface currents | 0.25° | Laurindo et al. 2017 |
| Zonal velocity variance | *Zvelv* | Measure of near surface eddy energy along Latitudes | 0.25° | Laurindo et al. 2017 |
| Meridional velocity variance | *Mvelv* | Measure of near surface eddy energy along Longitude | 0.25° | Laurindo et al. 2017 |
| Inflow from nearest channel | *Inflow* | Atlantic inflow (Sv) through the nearest channel | NA | Johns et al. 2002 |
| Distance to center of nearest channel | *Channel* | Distance to the center of the nearest channel. | NA | NA |
| Chlorophyll-a concentration | *Chla* | Chlorophyll-a concentration averaged over the previous three months | 0.036° | NOAA VIIRS |
| Windward | *Windward* | Binary predictor that reflects whether the location is leeward, east, (N) or windward (Y) of the Lesser Antilles island chain | NA | NA |
| Island | *Island* | Nearest island (categorical predictor) | NA | NA |

Table S2. Best variable combinations for each model type with associated QIC, AUC, goodness of fit and predictive accuracy (pre-cross validation).

| Model type | Variable type |  | QIC | AUC | Goodness of fit | Predictive accuracy  (±SE) |
| --- | --- | --- | --- | --- | --- | --- |
| Presence/absence | Environmental variable | Windward* + Chla + Shelf + Zvelv* + Inflow* + Channel + Canyon | 28541.9 | 0.765 | 68.98% | 32.73%  ±0.03 |
|  | Island | Windward + Island | 30684.9 | 0.690 | 65.82% | 59.61%  ±0.04 |
| EC1 | Environmental variable | Ecurr + Windward + Escarp + Abyss + Zvelv | 19006.3 | 0.786 | 77.14% | 56.65% ±0.03 |
|  | Island | Windward + Island | 15890.8 | 0.860 | 72.88% | 72.05%  ±0.04 |
| EC2 | Environment | Mvelv + Windward + Inflow* + Chla + Channel + Depth + Zvelv | 15710.9 | 0.879 | 74.55% | 37.63%  ± 0.03 |
|  | Island | Windward + Island | 16020.8 | 0.833 | 73.19% | 62.27%  ±0.04 |
| Vocal clan | Environment | Ecurr + Channel* + Zvelv | 5220.05 | 0.950 | 93.41% | 46.26%  ±0.03 |
|  | Island | Island | 1118.3 | 0.99 | 96.5% | 76.8%  ± 0.14 |

* Variables removed by stepwise cross-validation
